# Pyoderma Gangrenosum Induced by BNT162b2 COVID-19 Vaccine in a Healthy Adult

**DOI:** 10.3390/vaccines10010087

**Published:** 2022-01-07

**Authors:** Mazin Barry, Abdulaziz AlRajhi, Khaldoon Aljerian

**Affiliations:** 1 Division of Infectious Diseases, Department of Internal Medicine, College of Medicine, King Saud University, Riyadh 11451, Saudi Arabia; Dr.alrajhi@live.com; 2 Department of Pathology, College of Medicine, King Saud University, Riyadh 11451, Saudi Arabia; Kaljerian@ksu.edu.sa

**Keywords:** pyoderma gangrenosum, BNT162b2, mRNA, COVID-19 vaccine

## Abstract

(1) Background: Pyoderma gangrenosum (PG) is a rare neutrophilic dermatosis of unknown etiology. Coronavirus disease 2019 (COVID-19) vaccines can cause a variety of adverse cutaneous manifestations. PG associated with mRNA vaccines has not previously been described. This case study reports on the first patient to develop PG after receiving BNT162b2. (2) Case Presentation: An otherwise-healthy 27-year-old man developed multiple skin lesions 24 h after receiving the first dose of the messenger RNA-based Pfizer/BioNTech BNT162b2 COVID-19 vaccine. When in hospital, he developed a new painful ulcerative lesion on his right hand. Skin ulcer edge biopsy showed severe epidermal neutrophilic infiltrate with epidermal and dermal edema, underlying superficial dermal necrosis, and characteristic undermining with extensive mixed inflammatory infiltration of the dermis and abscess formation consistent with an ulcer with mixed dermal inflammation compatible with pyoderma gangrenosum. The lesion showed rapid improvement after the initiation of immunosuppressive therapy. (3) Conclusions: PG may be a rare adverse event related to the BNT162b2 vaccine, which could be more frequently encountered with the wide-scale use of mRNA vaccines. The continuous monitoring and surveillance of skin manifestations post-vaccination is essential.

## 1. Background

Coronavirus disease 2019 (COVID-19) has caused large disruptions to humanity and millions of deaths worldwide [1]. The rapid development of different COVID-19 vaccines has helped to drastically decrease the devastating burden of the pandemic. However, as with other vaccines, COVID-19 vaccines can cause a variety of adverse events, including pain or redness at the site of injection, fever, itching, chills, headache, muscle ache, fatigue, and joint pain [2]. Similarly, several cases of COVID-19 vaccine-induced skin changes have been reported, including urticaria, eczematous dermatitis, and granulomatous inflammation [3]. Pyoderma gangrenosum (PG) is a rare inflammatory skin disease characterized by painful pustules or nodules that ulcerate; it is thought to be immune induced by the improper function of neutrophils, leading to neutrophilic dermatosis and causing rapid development of painful cutaneous necrotic ulcerations [4]. In this case report, we present a young otherwise-healthy adult who developed pyoderma gangrenosum following the first dose of the Pfizer/BioNTech BNT162b2 COVID-19 vaccine.

## 2. Case Presentation

An otherwise-healthy 27-year-old man developed multiple painful skin swellings with redness that started after receiving the first dose of the BNT162b COVID-19 vaccine. The first painful swelling appeared on his lower right leg 24 h after vaccination; he visited a local hospital and was prescribed cephalexin for seven days. The swelling subsequently resolved. Four days after finishing the antibiotic course, he developed another painful swelling with redness over his lower left leg after minor blunt trauma. He visited a different local hospital, where he was prescribed another course of cephalexin for an additional seven days during which the swelling also resolved. Seven days later, he developed a new painful swelling involving his upper posterior right thigh extending into his peri-anal region; over the next three days, the pain increased and the swelling progressed, so he presented to our institution. He had no other symptoms, e.g., fever, joint pain, chest pain, palpitation, dyspnea, cough, sputum, loss of smell or taste, abdominal pain, or diarrhea. He reported no tick or insect bites, no exposure to animals, no high-risk sexual behavior, and no travel. He lives in Riyadh and works as a clerk. His past medical history revealed that four months prior to receiving the BNT162b2 vaccine, he presented to another hospital with a three-day history of pleuritic chest pain and shortness of breath two weeks after an upper respiratory tract infection (URTI). The hospital performed cardiac magnetic resonance imaging (MRI) and diagnosed him with myocarditis. The COVID-19 real-time reverse transcription polymerase chain reaction (RT-PCR) result from a nasopharyngeal swab (NPS) was negative at the time; however, no COVID-19 RT-PCR was performed two weeks earlier during the acute symptoms of the URTI. It is also noteworthy that he had no history of malignancy, autoimmune diseases, inflammatory arthritis, or inflammatory bowel disease.

On current presentation, the examination revealed that he was conscious, alert, and oriented, with a heart rate of 110 beat/minute, blood pressure of 120/80 mmHg, temperature of 37.1 °C, and respiratory rate of 16/minute. He appeared unwell. The right thigh examination showed a large erythematous area involving his right upper posterior thigh, which was warm, slightly raised, and tender. The peri-anal region was also erythematous, warm, and extremely tender. The lower legs were normal. The examination of the remaining skin did not reveal any rash or erythema. Joint, cardiovascular, and respiratory examinations were all normal. He had no palpable lymphadenopathy. His abdomen was soft, non-tender, and had no hepatosplenomegaly.

Laboratory investigations showed 120 g/dL hemoglobin (normal 125–170 g/L), 22,500/µL white blood cells (normal 4.0–11.0/µL) with 76% neutrophils, 267/µL platelets (normal 140–450/µL), a 87 mm/hour erythrocyte sedimentation rate (normal 0–17 mm/h), and 215 mg/L C-reactive protein (normal < 10 mg/L). Liver and renal function tests were normal. Antinuclear antibodies, antineutrophil cytoplasmic antibodies, prothrombin time, activated partial thromboplastin time, fibrinogen and D-dimer were also all normal. *Brucella* antibodies and human immunodeficiency virus serology were negative, the NPS for severe acute respiratory syndrome coronavirus 2 (SARS-CoV-2) RT-PCR was negative, and qualitative chemiluminescence immunoassay SARS-CoV-2 S-RBD IgG was positive at 3.71 AU/mL (normal range 0.00–0.99 AU/mL). A computed tomography scan of the pelvis revealed right peri-anal focal hypodensity measuring 2.4 × 1.3 cm associated with diffuse skin thickening and subcutaneous edema, suggestive of a small peri-anal collection. He was admitted to hospital and started on intravenous piperacillin/tazobactam and vancomycin. On day one of his hospital stay, he underwent the incision and drainage of the peri-anal area, a small amount of pus was drained, and *Klebsiella pneumoniae* and *Pseudomonas aeruginosa* that were both susceptible to piperacillin/tazobactam grew in culture; vancomycin was stopped on day four of his hospital stay.

**Figure 1:**
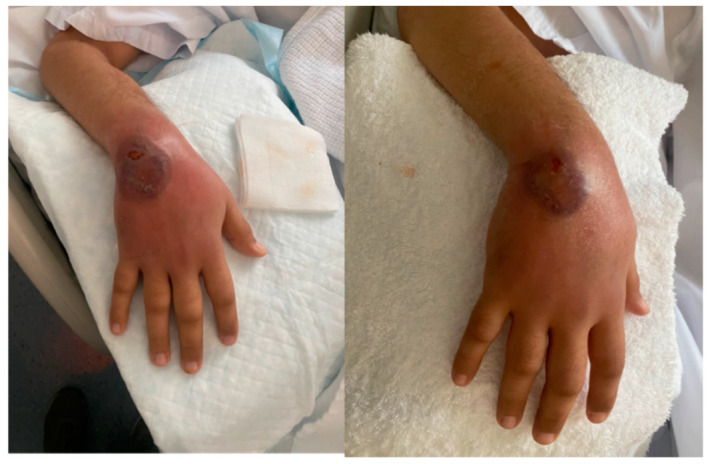
Right hand swelling with eschar on the dorsum that developed in hospital.

**Figure 2:**
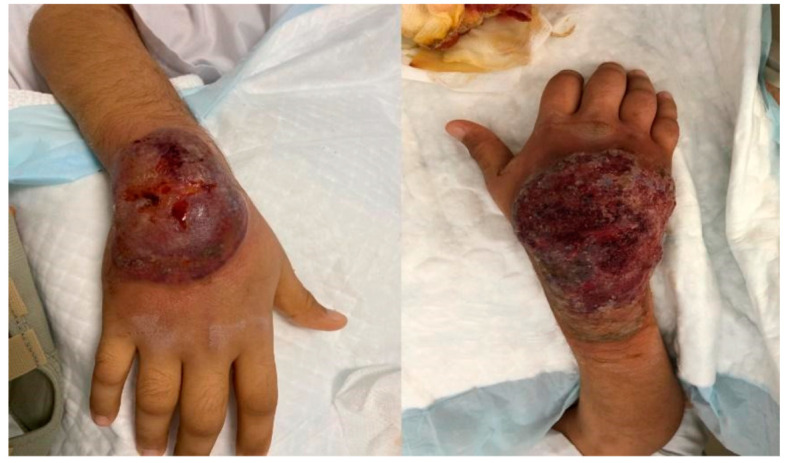
Progression and ulceration of lesion two days after appearance.

**Figure 3:**
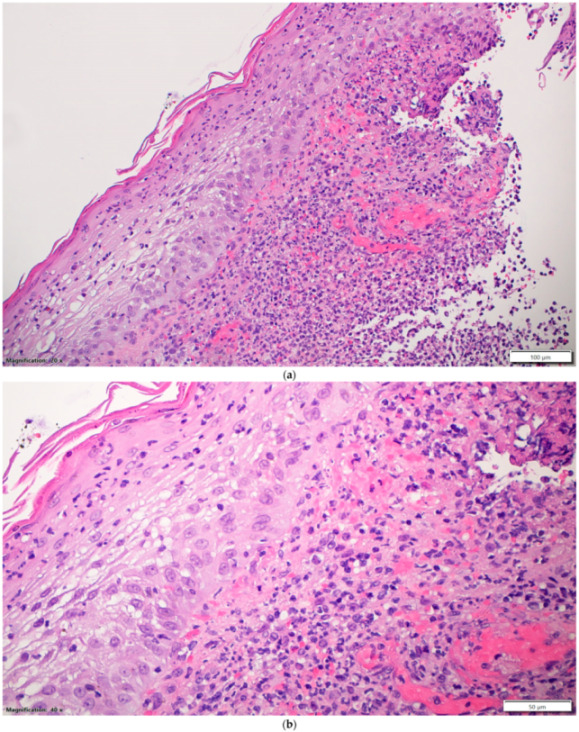
Epidermal neutrophil infiltration and underlying abscess formation and hemorrhagic foci: (**a**) hematoxylin and eosin stain (H&E) 20× objective; (**b**) H&E 40× objective.

**Figure 4:**
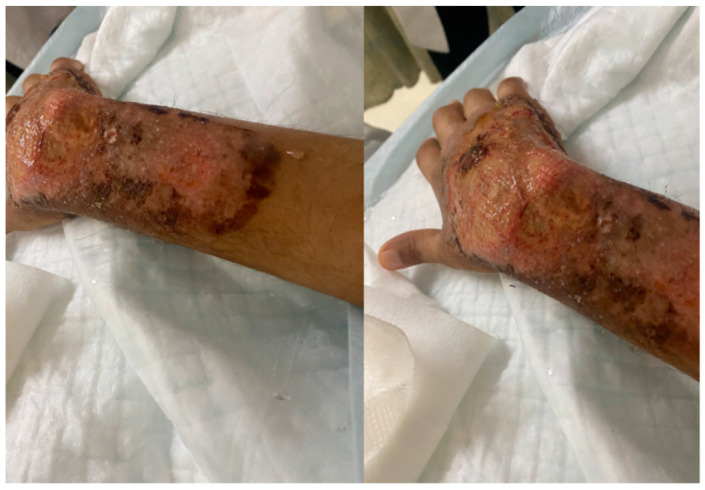
Resolution of swelling and eschar post treatment.

On day 14 of his hospital stay, he developed a new painful swelling overlying his right hand (Figure 1). Examination revealed moderate swelling of the dorsum of the right hand with a black eschar overlying his wrist with severe tenderness. Two days later, on day 16 of hospital stay, it became ulcerated (Figure 2). Antibiotics were stopped, and 24 h later, multiple punch biopsies were taken from the ulcer edge. The specimen was fixed for 48 h in 10% buffered formalin. The tissue sample was then sectioned and placed in a histology cassette for processing. The samples were then embedded in paraffin and sectioned (2–3 μm). Tissue sections were stained with hematoxylin and eosin (H&E) for histopathological examination by light microscopy. Microscopy revealed a severe epidermal neutrophilic infiltrate with epidermal and dermal edema. There was underlying superficial dermal necrosis, and characteristic undermining with extensive mixed inflammatory infiltration of the dermis and abscess formation and hemorrhagic foci (Figure 3a,b). Gram, acid-fast bacilli and fungal stains did not show any organisms. *Mycobacteria tuberculosis* PCR was non-reactive. Tissue cultures did not grow any bacterial organisms. Fungal and mycobacterial cultures ultimately did not grow neither. The patient was diagnosed with pyoderma gangrenosum (PG) based on the clinical presentation and histopathology, and on day 24 of his hospital stay, he was treated with prednisone 1 mg/kg IV daily, which resulted in the rapid improvement of his pain, swelling, erythema, and ulcer within three days (Figure 4). He was discharged from hospital on day 28 of his hospital stay on a tapering course of oral prednisone. Three weeks later, he visited the outpatient clinic with complete resolution of the lesion and no further recurrent episodes.

## 3. Discussion

This case report presents a young otherwise-healthy adult who developed pyoderma gangrenosum (PG) following his first dose of the BNT162b2 COVID-19 vaccine. It is likely that his myocarditis that was attributed to a viral infection four months before he received the vaccine was possibly due to undiagnosed COVID-19, hence inducing a strong immune response when challenged with the vaccine, which may have triggered his PG. This hypothesis may be further supported by his reactive anti-SARS-CoV-2 S-RBD IgG assay result, which is more likely to be reactive due to his past infection rather than a single dose of the vaccine, though the combination of both would also elicit a positive response.

PG is an inflammatory skin condition that is both rare and difficult to diagnose. Therefore, a group of international experts in 2018 published diagnostic criteria based on a Delphi consensus exercise using the RAND/UCLA Appropriateness Method [5]. The result revealed one major criterion and eight minor criteria and found that four of eight minor criteria maximized discrimination, which resulted in 86% sensitivity and 90% specificity [5]; our patient fulfilled all the major criteria and all but one of the minor criteria (Table 1).

Magro et al. studied the biopsies of 22 patients who developed adverse skin changes after receiving mRNA-1273 or BNT162b2 vaccines [3]. They reported interface dermatitis, eczematous dermatitis, lymphocytic vasculitis, Grover’s disease, urticarial vasculitis, urticaria, perniosis, and granulomatous inflammation, with one case exhibiting an interstitial pattern and another exhibiting a folliculocentric neutrophilic and granulomatous reminiscent of vesiculopustular pyoderma gangrenosum [3]. Most of the cases reported in the same study demonstrated type IV hypersensitivity reaction and less commonly that of immune-complex-mediated hypersensitivity [3]. Moreover, Ramessur et al. reported a 73-year-old man who developed cutaneous thrombosis associated with skin necrosis following the first dose of the vector-based ChAdOx1 nCov-19 vaccine [6].

A registry-based study in USA collected cases of cutaneous manifestations after mRNA COVID-19 vaccination; it identified 414 reactions, mostly due to mRNA-1273 in 83% of cases, and only 17% occurring with BNT162b2, with most manifesting as local reactions followed by urticarial eruptions and morbilliform eruptions [7]. They also rarely reported pernio/chilblains, cosmetic filler reactions, zoster, and pityriasis rosea-like reactions, but no PG [7]. Similarly, Sweet syndrome is a rare neutrophilic dermatosis affecting all layers of the skin with or without true vasculitis and most commonly idiopathic, but several drugs have been reported to induce it, and it was recently reported in a 52-year-old Caucasian man three days after receiving the first dose of the BNT162b2 vaccine [8]. Several theories regarding its pathogenesis have emerged, including hypersensitivity to bacterial or viral antigens that may trigger neutrophilic activation and subsequent infiltration [9]. Similar triggering caused by an mRNA vaccine or one of its components is plausible.

Evidence that accumulated during the COVID-19 pandemic has drawn attention to neutrophils’ transcriptional and functional diversity and their important role in the pathophysiology [10]. In addition, there is generation of neutrophil extracellular traps and the presence of immature, immunosuppressive, and activated neutrophil subsets in the circulation, and a granulocytic signature is emerging as a defining feature of severe COVID-19 [11]. Moreover, mRNA can serve as both immunogen and adjuvant as a critical part of the innate immune response to viruses [12]. Furthermore, a recent study reported a 71-year-old-man with type 2 diabetes mellitus, who developed PG ten days after he was diagnosed with COVID-19 [13].

Although PG being induced by vaccination is extremely rare, it has been reported in an otherwise-healthy 72-year-old man 12 h after the influenza vaccine, who developed neutrophilic dermatosis [14]. Similarly, our case developed PG 24 h after vaccination.

## 4. Conclusions

This is the first reported case of pyoderma gangrenosum induced by the BNT162b2 COVID-19 vaccine.

The temporal relationship between receiving the vaccine and development of the patient’s first lesions strongly supports its etiological role. With the increasing uptake of mRNA-based vaccines, clinicians should be aware of the diversity of adverse events that may be encountered, including unusual skin manifestations. The ongoing surveillance and reporting of various cutaneous adverse events is crucial.

## Figures and Tables

**Table 1:** Delphi criteria for pyoderma gangrenosum diagnosis with corresponding fulfillment of current patient.

Type of Pyoderma Gangrenosum Diagnostic Criteria	Description	Patient Fulfillment
Major criterion	Biopsy of ulcer edge demonstrating neutrophilic infiltrate	Yes
Minor criterion	Exclusion of infection	Yes
Minor criterion	Pathergy	Yes
Minor criteria	History of inflammatory bowel disease or inflammatory arthritis	No
Minor criteria	History of papule, pustule, or vesicle ulcerating within 4 days of appearing	Yes
Minor criteria	Peripheral erythema, undermining border, and tenderness at ulceration site	Yes
Minor criteria	multiple ulcerations, at least 1 on an anterior lower leg	Yes
Minor criteria	Cribriform or “wrinkled paper” scar(s) at healed ulcer sites	Yes
Minor criteria	Decreased ulcer size within 1 month of initiating immunosuppressive medication(s)	Yes

## Data Availability

Not applicable.

## Abbreviations

PG	pyoderma gangrenosum
COVID-19	coronavirus disease 2019
mRNA	messenger ribonucleic acid
URTI	upper respiratory tract infection
MRI	magnetic resonance imaging
NPS	nasopharyngeal swab
RT-PCR	reverse transcription polymerase chain reaction
SARS-CoV-2	severe acute respiratory syndrome coronavirus 2
H&E	hematoxylin and eosin
RAND	research and development
UCLA	University of California at Los Angeles
